# Comparative Genomics of Two New HF1-like Haloviruses

**DOI:** 10.3390/genes11040405

**Published:** 2020-04-08

**Authors:** Mike Dyall-Smith, Sen-Lin Tang, Brendan Russ, Pei-Wen Chiang, Friedhelm Pfeiffer

**Affiliations:** 1 Computational Biology Group, Max-Planck-Institute of Biochemistry, 82152 Martinsried, Germany; fpf@biochem.mpg.de; 2 Veterinary Biosciences, Faculty of Veterinary and Agricultural Sciences, University of Melbourne, Parkville 3010, Australia; 3 Biodiversity Research Center, Academia Sinica, Nankang, Taipei 115, Taiwan; sltang@gate.sinica.edu.tw (S.-L.T.); chiangpw@gate.sinica.edu.tw (P.-W.C.); 4 Department of Microbiology, Monash University, Clayton Campus, Victoria 3800, Australia; brendan.russ@monash.edu

**Keywords:** archaea, haloarchaea, halovirus, virus genome, *Halorubrum*, *Haloferax*

## Abstract

Few genomes of the HF1-group of viruses are currently available, and further examples would enhance the understanding of their evolution, improve their gene annotation, and assist in understanding gene function and regulation. Two novel HF1-group haloviruses, Serpecor1 and Hardycor2, were recovered from widely separated hypersaline lakes in Australia. Both are myoviruses with linear dsDNA genomes and infect the haloarchaeon *Halorubrum coriense*. Both genomes possess long, terminal direct repeat (TDR) sequences (320 bp for Serpecor1 and 306 bp for Hardycor2). The Serpecor1 genome is 74,196 bp in length, 57.0% G+C, and has 126 annotated coding sequences (CDS). Hardycor2 has a genome of 77,342 bp, 55.6% G+C, and 125 annotated CDS. They show high nucleotide sequence similarity to each other (78%) and with HF1 (>75%), and carry similar intergenic repeat (IR) sequences to those originally described in HF1 and HF2. Hardycor2 carries a DNA methyltransferase gene in the same genomic neighborhood as the methyltransferase genes of HF1, HF2 and HRTV-5, but is in the opposite orientation, and the inferred proteins are only distantly related. Comparative genomics allowed us to identify the candidate genes mediating cell attachment. The genomes of Serpecor1 and Hardycor2 encode numerous small proteins carrying one or more CxxC motifs, a signature feature of zinc-finger domain proteins that are known to participate in diverse biomolecular interactions.

## 1. Introduction

The closely related archaeal haloviruses HF1 and HF2 were first described 27 years ago [1], and are lytic, tailed viruses belonging to the family *Myoviridae*. Isolated from the same saltern crystallizer in Geelong, on the south-eastern coast of Australia, they infect distinct species of haloarchaea belonging to different genera, *Haloferax lucentense* (HF1) and *Halorubrum coriense* (HF2). They share similar virion morphologies and their linear dsDNA genomes are closely related, of similar length (75,898 and 77,672 bp, respectively), and possess long terminal direct repeats (TDR) of 306 bp [2,3,4]. The replication strategy of HF2 was reported by Nuttall and Dyall-Smith [2], who detected concatemeric forms of the genome and a potential nick recognition sequence at the TDR border. The transcription program of HF2 was described by Tang et al. [4] and found to be divided into three main phases: immediate-early (0–1 h p.i.), middle (1–3 h p.i.) and late (3–5 h p.i.). These phases corresponded to consecutive regions of transcription of the genome (approximately 0–5 kb, 5–40 kb and 40–76 kb, respectively). 

The genome sequence of HF2 was reported in 2002 [4], and two years later this could be compared to the genome of HF1 [3]. Surprisingly, their sequences are almost identical over the first 48 kb (ORFs 1–83) but from there onwards they diverge considerably (85% nucleotide similarity). This more diverged region covers the late transcription region carrying genes involved in DNA packaging, virus morphogenesis and assembly. The stark change in sequence similarity indicated that a recombination event had occurred, and the replaced region provided a good explanation for the different host ranges of the two viruses, since the late region encompasses genes for virion assembly which must include the receptor binding domains (RBDs). In similar myoviruses, RBDs are commonly borne by tail fiber proteins [5], directing them to bind specifically to the host cell surface. The two genomes also revealed a number of prominent AT-rich intergenic repeat (IR) sequences that were located in strategic positions relative to the mapped transcripts (see Figure 1 of [4]), and each contained functional promoter elements (Russ et al., manuscript in preparation).

In 2012, the tailed haloviruses HRTV-5, HRTV-7 and HRTV-8 were reported [6]. All three infected strains of *Halorubrum*. These are also myoviruses with linear, dsDNA genomes, and show sequence similarity to the genomes of HF1 and HF2 [7,8]. In the current study, the genomes of two additional HF1-group viruses, Serpecor1 and Hardycor2, were sequenced, annotated and compared to the five related viruses as well as to two proviruses, Hdep-prov1 and ELPmg-prov1, one present in the genome of *Halorubrum depositum* and the other found in a metagenomic contig recovered from Eden Landing (salt) Ponds, USA. In addition, mass spectrometry and protein sequencing of the major virion proteins of HF2 identified their corresponding genes. Finally, a combination of host range data and comparative genomics allowed the probable RBDs of these viruses to be identified.

## 2. Materials and Methods 

### 2.1. Virus Isolation and DNA Sequencing

Hardycor2 and Serpecor1 were recovered from hypersaline waters sampled in 1998 in Australia. They were isolated from Lake Hardy, Victoria (35°3’58.27" S 141°44’12.50" E) and Serpentine Lake, Western Australia (32°0’16.14" S, 115°31’34.01" E), respectively. Isolation methods and conditions were as described previously for halovirus SH1 [9] except that the isolating host was *Halorubrum coriense*, cultivated at 37 °C. Soft-agar overlay plates were incubated for 48–72 h until plaques became visible. After three rounds of plaque purification, virus stocks were stored in HF diluent [10] at −80 °C.

### 2.2. Electron Microscopy

Samples were negatively stained with 2% uranyl acetate, and examined on Formvar-coated copper grids (200 mesh) using a JEOL 1200EX JEM electron microscope (JEOL Ltd., Tokyo, Japan), at 50,000× magnification, and 80 kV.

### 2.3. Halovirus HF2 Protein Analyses

Large-scale growth of HF2 in *Hrr. coriense* cultures and purification on CsCl gradients was performed as described previously [2,4,10]. Approximately 10^11^ pfu of purified virus was treated with trichloroacetic acid (10% (*v*/*v*) final concentration) to precipitate proteins, which were collected by centrifugation (16,000·*g*, 15 min), washed twice with acetone, then air-dried before dissolving in Laemmli sample buffer [11] with 48 mM *β*-mercaptoethanol. Samples were heated in boiling water for 5 min, then separated on a 12% (*w*/*v*) NuPAGE Bis-Tris Gel (Invitrogen/Novex, Waltham, USA) using MES-SDS running buffer, according to the manufacturer’s instructions (Invitrogen, Waltham, USA). After electrophoresis, gels were rinsed in double-distilled H_2_O and stained with 0.1% (*w*/*v*) Coomassie Brilliant Blue G in methanol:acetic acid (40% (*v*/*v*) and 10% (*v*/*v*), respectively), and destained in the same methanol:acetic acid mixture but without stain. The correction formula of Guan et al. [12] was used to estimate the apparent molecular weights of HF2 proteins separated by SDS–polyacrylamide gel electrophoresis. Protein bands were then excised and sent to the Australian Proteome Research Facility (APRF), Macquarie University, for trypsin digestion and analysis by matrix-assisted laser desorption ionization time of flight mass spectrometry (MALDI-TOF MS) on an Applied Biosystems 4700 Proteomics Analyser (Applied Biosystems, Waltham, USA). N-terminal amino acid sequencing was also performed at the APRF.

### 2.4. DNA Sequencing 

Virus stocks that had been stored frozen at −80 °C since 1999 were thawed, and total DNA was extracted from each using the CTAB/phenol–chloroform procedure [13]. After quality and quantity checks of the DNAs by agarose gel electrophoresis, the total DNA was amplified using the Qiagen REPLI-g Mini Kit (QIAGEN Inc., Valencia, USA) in order to achieve sufficient quantities of DNA for next-generation sequencing. The amplified DNAs were checked using agarose gel electrophoresis and purified using QIAamp^®^ DNA Mini Kits (QIAGEN Inc., Valencia, USA). DNA concentrations were determined using NanoDrop 1000 (Thermo Scientific, Carlsbad, USA) and Qubit (Invitrogen) instruments. One microgram from each DNA preparation was sent to the Yourgene Health Co. (Taipei, Taiwan) for sequencing using the Illumina platform (HiSeq 2500 sequencer (Illumina, San Diego, USA); 2 × 250 bp paired-end reads).

### 2.5. Assembly and Bioinformatics Analyses

Assembly of Illumina reads was performed using the de novo assembler within Geneious version 10.2.6 (https://www.geneious.com) [14]. The Yass aligner [15] for DNA dot plots was accessed via the online webserver (https://bioinfo.lifl.fr/yass/yass.php) and used with an E-value setting of ≤10^−4^. Multiple alignment used the CLUSTAL Omega aligner (https://www.ebi.ac.uk/Tools/msa/clustalo/) [16]. Phylogenetic tree reconstructions from protein sequences used the Genome-BLAST Distance Phylogeny method (GBDP) under optimal settings (formula VICTOR *d6*), as implemented at the DSMZ webserver https://victor.dsmz.de. Branch lengths were scaled in terms of the GBDP distance formula *d6* [17].

## 3. Results

### 3.1. Virus Isolation 

**Figure 1:**
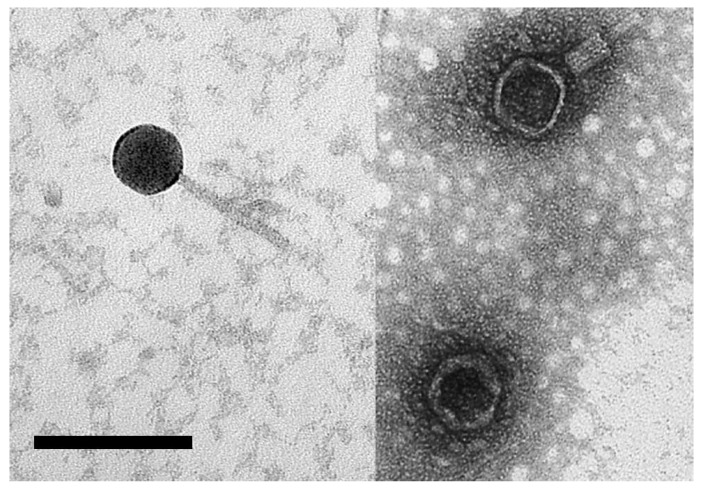
Electron-micrograph of halovirus Serpecor1. Negatively stained with 2% uranyl acetate. Size bar, 100 nm

Samples from the hypersaline waters of Lake Hardy (Victoria) and Serpentine Lake (Western Australia) were mixed with *Hrr. coriense* cells, plated as soft-agar overlays, and incubated for 48–72 h. A clear plaque from each water sample was picked, and plaque-purified three times. Originally, the two virus isolates were labeled HC2 and SC1, but are now renamed Hardycor2 and Serpecor1, reflecting their origin (Hardy/Serpentine lakes) and host species (*Hrr. coriense*). Both viruses consistently gave clear plaques, with diameters of 3–5 mm. Negative-stain electron microscopy of Serpecor1 (Figure 1) revealed a myovirus-like morphology (head diameter, 46 nm; tail length, 80 nm). A virus particle with a contracted tail can be seen in the upper right. Hardycor2 gave such low titres that no particles were detected by electron microscopy.

### 3.2. DNA Sequencing and Annotation

The sequence reads obtained from the DNAs of the two virus stocks were assembled into high-coverage contigs spanning each halovirus genome (Table 1). 

Both genomes are linear dsDNA, similar in length and %G+C (56–57%), and contain long terminal direct repeats (TDRs); 320 bp for Serpecor1 and 306 bp for Hardycor2. Initial BLASTn comparisons showed that not only were the two viruses closely related to each other, they were also closely related to haloviruses HF1, HF2, HRTV-5, HRTV-8 and more distantly related to HRTV-7. Searches of the GenBank database (BLASTn) identified two proviruses, denoted ELPmg-prov1 and Hdep-prov1 (Table 2), that were also similar to Serpecor1 and Hardycor2, and they are included in the following comparative analyses. For convenience, all of these related viruses and proviruses will be referred to as the HF1-group. They are all myoviruses with linear, dsDNA genomes that carry long TDRs [3,7], and their general properties are summarized in Table 2.

**Figure 2:**
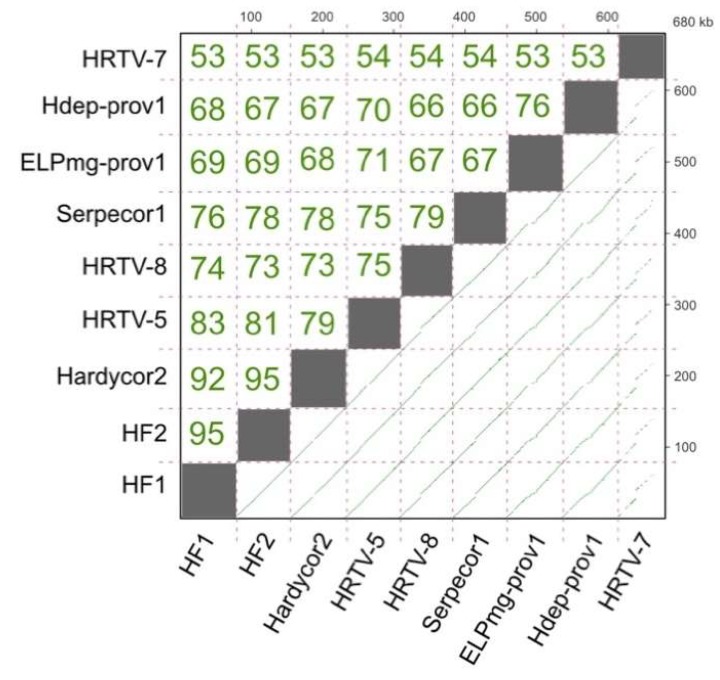
Nucleotide similarity between the genomes of Serpecor1, Hardycor2 and the HF1-group of viruses, including proviruses Hdep-prov1 and ELPmg-prov1. The lower right triangle shows the dot plots of DNA sequence similarity using the Yass aligner [15], where green lines show similarity at a BLASTn *E*-value setting of ≤ 10^−20^). The cumulative length of DNA is shown by the scales on the right and upper axes, in kb. Upper triangle shows the pairwise similarity values (percentage of identical nucleotides) after multiple alignment of all genomes using the MUSCLE aligner option within Geneious.

Genome length varies between 74.2–77.6 kb, except for HRTV-7 which is significantly shorter at 69.0 kb. A dot plot of the nucleotide similarity of all HF1-group viruses is presented in Figure 2, along with their pairwise similarity values calculated after multiple alignment. All but HRTV-7 have similarity values between 66% and 95%. Hardycor2 shows the highest similarity to HF1 and HF2 (92–95%), while Serpecor1 is most similar to HRTV-8 (79%). The outlier is HRTV-7, which shows 53–54% nucleotide similarity to all other members of the HF1-group, and only patchy lines of similarity in dot plot comparisons.

Multiple alignment of the HF1-group genomes led to the identification of a few errors in the available genome sequences of HF1 and HF2, and, by re-examining the original Sanger reads, these could be corrected (two base differences for HF1, four base differences for HF2; Table S1). The corrected sequences and revised annotations have been submitted to GenBank and the updated versions are now available as AY190604.2 and AF222060.2. HF1 has 125 annotated CDS, and HF2 now has 126 CDS. 

The avoidance of palindromic motifs such as GATC and its inverse CTAG was first noted in the genomes of HF1 and HF2 [3,4], and provides protection against anti-viral defenses of the host that target palindromic recognition sites. Both *dam* (GATC) and *zim* (CTAG) restriction-modification (RM) systems have been described in *Haloferax* [20,21,22]. Tetramer frequency analysis of all HF1-group genomes (Table 3) revealed the widespread under-representation of GATC and CTAG across all examples, with these being completely absent in all seven virus isolates and one provirus (ELPmg-prov1), while in the other provirus (Hdep-prov1) there are only three CTAG sites. GATC is reduced to 0.45 of the expected frequency in both proviruses. The motif AGCT is absent in the HRTV-7 genome. The motifs TGCA and CATG are under-represented in all cases, and this is even more evident when longer palindromes containing these core motifs are examined (highlighted in Tables S2 and S3). Excluding longer motifs that contain CTAG/GTAC, twenty-one 6-mer target palindromes were found to be absent across all HF1-group genomes (Table S2), and another twenty 6-mer palindromes were present in only one or two viruses, and then usually at very low frequencies (Table S3). TTCGAA is a target motif for methylation in *Halobellus limi* [23], and is absent in all HF1-group genomes except for a single site in Hdep-prov1. Even the 5-mer sequence GGWCC, a methylation motif reported in *Salarchaeum* sp. JOR-1 [24], is absent in all HF1-group viruses except HRTV-7.

Genome annotation revealed 126 (Serpecor1) and 125 (Hardycor2) CDS. Hardycor2 had two predicted tRNA genes: tRNA-Arg(TCT) and tRNA-Asn(GTT), while Serpecor1 had only the tRNA-Asn(GTT). Curiously, Serpecor1 has a partial tRNA-Arg(TCT) found in the same position as in Hardycor2 but with a 28 nt deletion. The same deletion also occurs in HRTV-8. A partial tRNA-Thr(GGT) sequence located near to a site-specific integrase (e.g., HfxHF1_440) is found in all HF1-group viruses, including the proviruses Hdep-prov1 and ELPmg-prov1 (see later). The conserved position next to the integrase suggests that it can act as an *attP* element for the integration of the virus genome into a host tRNA gene, and this is indeed observed to be the case for Hdep-prov1 in the genome of *Hrr. depositum* (see later). The tRNA prediction algorithm (tRNAscan) also flagged a putative tRNA- Pro(TGG) upstream of the large subunit terminase gene (HfxHF1_615), and although it is conserved across all HF1-group genomes it has an aberrant secondary structure and a sequence that is not closely similar to known tRNAs. Since HRTV-7 is distinctly less related to the other HF1-group viruses, and is difficult to align with them at the nucleotide level, it will not be included in the comparative analyses described in the following sections.

In tailed viruses, there are often two small, overlapping CDS coding for chaperones found immediately upstream of the gene for the tape measure protein that are translated as a single protein via programmed translational frameshifting [25]. A similar gene arrangement is present in the HF1 group, where there are two CDS annotated between the genes for the tail tube and tape measure proteins. The alignment of the six HF1 group genomes shows high sequence conservation near the end of the first CDS, and a translational fusion with the downstream CDS would require a +1 translational frameshift, but conventional slippery sequences appear to be absent. However, very near the stop codon of the first CDS is a TTT-CGC motif that lies within a perfectly conserved 15 bp region, and such a motif (TTT-CGn) has been implicated in +1 frameshifts in a number of eukaryotic viruses [26,27]. Frameshifting may be enhanced by pausing events caused by the surrounding sequence, specific tRNA interactions or codon frequency [26]. In *Halorubrum*, TTT codons are rare (3.1 per 1000 codons), and the final AGA codon (Arg) of the first CDS is even less common (1.8 per 1000 codons (https://hive.biochemistry.gwu.edu/cuts/) [28]. Experimental evidence will be needed to unravel the details of the translational frameshifting involved. 

### 3.3. Identity of the Genes Encoding the Major Structural Proteins of HF2

**Figure 3:**
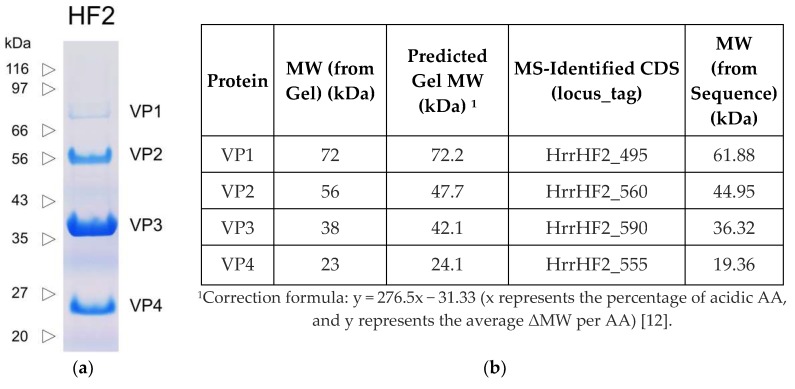
(**a**) SDS-PAGE of HF2 proteins (Coomassie Blue stained). (**b**) Molecular weights and identified genes of VP1-VP4.

The proteins of purified HF2 were separated by SDS-PAGE and stained with Coomassie Brilliant Blue (Figure 3). Four major bands (VP1-VP4) were detected, with apparent molecular weights of 23–72 kDa (Figure 3b). To identify the proteins in these bands, they were excised and analyzed by mass spectrometry (Figure S1), and their corresponding locus tag is given in Figure 3b. The N-terminal sequence of VP3 was determined to be VNRDI, which corresponds to amino acids 2-6 of HrrHF2_590. Since the codon for the valine is GTT and not a potential start codon, the results are consistent with the annotated methionine initiator being post-translationally removed. The same processing event has been described for the major capsid protein (gp13) of halovirus HSTV-2, a myovirus with a similar particle morphology, genome organization and MCP (43% aa identity) as HF2 [29].

### 3.4. Comparative Genomics

**Figure 4:**
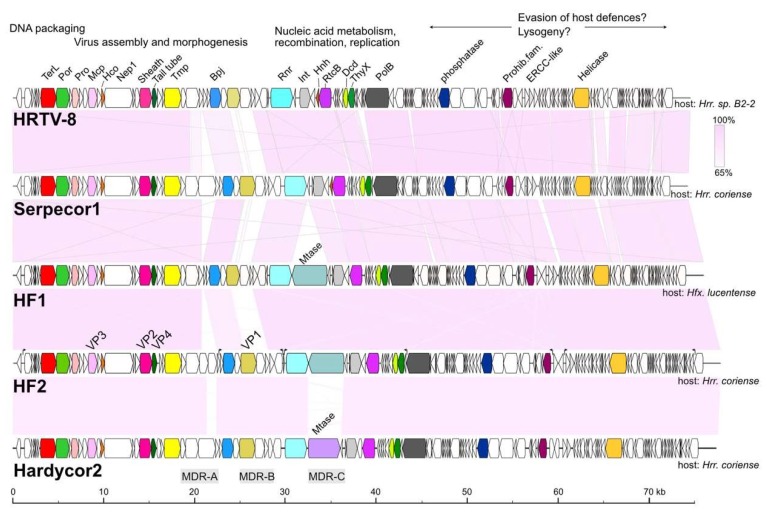
Genome maps of haloviruses Serpecor1 and Hardycor2 along with their closest relatives, HF1, HF2 and HRTV-8. Pink shading between the maps indicates nucleotide similarity values above 65% (scale at top right). Virus names are given at the left, host species are indicated at the right, and protein names are given at the top of HRTV-8, near the corresponding colored CDS. Corresponding genes on each map have the same color. Scale at bottom represents DNA length in kb. At top are headings indicating the known or likely functional modules (DNA packaging, Virus assembly, etc.). Major Divergent Regions (MDR) are indicated below the Hardycor2 map. Protein names are: TerL, large subunit terminase; Por, portal protein; Pro, prohead protease; Mcp, major capsid protein; Hco, head connector protein; Nep1, neck protein of type 1; Sheath, tail sheath protein; Tail tube, tail tube protein; Tmp, tape measure protein; Bpj, baseplate J family protein; Rnr, ribonucleotide reductase; Int, site-specific integrase; Hnh, HNH-endonuclease; RtcB, tRNA splicing ligase RtcB; Dcd, dCTP deaminase; ThyX, thymidylate synthase; PolB, DNA polymerase elongation subunit (family B); Prohib. fam., prohibitin family protein; ERCC-like, ERCC nuclease family; Helicase, ATP-dependent DNA helicase; Mtase, methyltransferase. The virus structural proteins of HF2 (VP1–VP4) are indicated just above the HF2 genome map.

The genome maps of Serpecor1 and Hardycor2 are shown in Figure 4, along with maps of their closest relatives, HF1, HF2 and HRTV-8. All have TDR (Table 2) and corresponding genes are colored the same on different genomes. The light pink shading between maps indicates high nucleotide similarity, which is also reflected by the strongly conserved gene synteny among these viruses. Three major variable regions, MDR-A, -B and -C (interrupted shading), are found within a roughly 16 kb region between the genes encoding tape measure protein (Tmp) and the site-specific integrase (Int), or from about 20 to 36 kb in Figure 4. Variation in this region was first reported between HF1 and HF2 (see Figure 1 of [3]); two viruses that share high sequence similarity but have distinctly different host ranges. Another notable difference seen in Hardycor2 is the gene (HrrHc2_200) for a methyltransferase (Mtase). Five viruses (HF1, HF2, HRTV-5, ELPmg-prov1 and Hdep-prov1) carry an N-6 DNA methyltransferase in the same gene neighborhood, and the encoded proteins share high sequence similarity (78–96% aa identity, Figure S1), however, the Hardycor2 methyltransferase is only distantly related to these (16% aa identity) and the gene is oppositely oriented (Figure 4), indicating a distinct evolutionary history. Serpecor1 and HRTV-8 lack a methyltransferase gene at this position, highlighting the flexibility for gene insertion and loss in this region, perhaps influenced by the nearby integrase and *attP*. Two types of long, AT-rich, intergenic repeat (IR) sequences, designated class I and class II, were described in HF1 and HF2 [3,4] and speculated to control the transcription of the genome. These IRs are strongly conserved among HF1-group viruses, and a comparison between the IR sequences of HF2 and those of Hardcor2 and Serpecor1 is given in Supplementary Figure S3. The promoter activities of these motifs in the HF2 genome have been confirmed [30] (Russ et al., manuscript in preparation).

A significant proportion of genes of the HF1-group viruses code for proteins containing one or more CxxC motifs, which are signature features of zinc-finger (ZF) domains. In general, ZF domains predominantly function as interaction modules that can bind to nucleic acids, proteins and other small molecules, including lipids [31,32]. Serpecor1 carries 23 annotated genes specifying CxxC motif proteins, and Hardycor2 carries 21 such genes. While two of the larger proteins in this group (ribonucleotide reductase and Rad3-related helicase) are known to bind nucleic acids, the majority of CxxC motif proteins are small (micro-) proteins, less than 100 aa (14/23, Serpecor1;12/21, Hardycor2) with unknown or poorly understood functions. The gene distribution for these CDS is uneven in both genomes. For example, none occur in the 26.4 kb region from *terL* to the gene preceding that for ribonucleotide reductase (Rnr) in both viruses, a region involved in viral assembly and morphogenesis, which includes all the genes for virus structural proteins (Figure 4).

### 3.5. HF1-Group Proviruses

Two HF1-like provirus genomes (Hdep-prov1 and ELPmg-prov1) were retrieved by BLASTn searches of the GenBank database using HF1 as the query (Table 2). Hdep-prov1 is a 77.6 kb element present in the genome of *Hrr. depositum* Y78, is flanked by two annotated tRNA-thr genes (FGM06_RS03355 and FGM06_RS03870), and has an integrase gene close to one end (FGM06_RS03360). The second provirus we designated ELPmg-prov1, and was described as part of a metagenomic study of the Eden Landing Ponds, San Francisco, USA [19]. It is 77.7 kb long, has an integrase gene near one end, and has recombined into a tRNA-Thr gene present on an 82.4 kb contig. To identify the likely host species, the two flanking sequences of the contig were used to search the GenBank database (BLASTn), and both sequences matched *Halobacterium hubeiense* as the top hit (90–94% nucleotide identity; data not shown). These proviruses display the typical features of a temperate virus that has integrated into the host chromosome via recombination at a tRNA gene [33]. 

**Figure 5:**
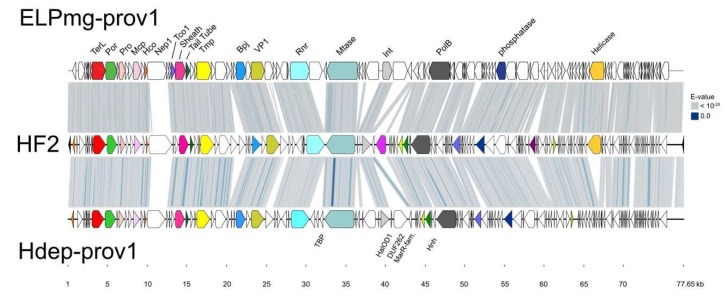
Comparison of HF2 and the provirus elements Hdep-prov1 of *Hrr. depositum* Y78 (located between FGM06_RS03355 to FGM06_RS03870) and ELPmg-prov1 (from scaffold ID JGI12451J12833_1000001; NCBI project accession PRJNA366386). Scale is shown underneath, in kb. The tBLASTx similarity between the genomes is indicated by the grey-to-blue shading (key upper right). A number of gene products are indicated above the ELPmg-prov1 map (see Figure 4 for details). Additional genes are indicated below the Hdep-prov1 map as follows; TBP, transcription factor B protein; HalDo1, Halobacterial output domain 1 containing protein; DUF262, domain of unknown function (DUF) 262 containing protein; MarR-fam., MarR family transcription factor; Hnh, HNH endonuclease.

Both sequences were circularized at their *att* sites, re-opened at the probable terminus sequence (TDR) of the linear dsDNA virion genome, and aligned to HF2 (Figure 5). They share 67–69% nucleotide similarity with HF2 (Figure 2), and the maps reveal the close synteny with HF2. Differences are evident from the absence of shading (tBLASTx similarity) between the genome maps, and include the MDR-A, -B and -C regions described earlier, but also a number of specific gene differences. For example, the long, divergent gene specifying Nep1 that lies within the virus assembly module (uncoloured, at around 10–12 kb of Figure 5). Curiously, the two provirus *nep1* genes are similar to the first 387 nt of the corresponding HF2 gene (HrrHF2_575) but not to the remaining 2724 nt. Over the region of similarity, the inferred proteins of the proviruses are 79% identical to the corresponding HF2 protein, and it is within this initial 129 aa that the VIRFAM database (http://biodev.cea.fr/virfam) detects a significant match to the HK97 gp10 family phage proteins, which they name Ne1 [34] and we refer to as Nep1. In the right half of Figure 5, both proviruses are seen to lack genes for the RNA-splicing ligase RtcB (HrrHF2_430; magenta) and prohibitin family protein (HrrHF2_235; dark purple), while the gene for the Rad3-related helicase (HrrHF2_140; amber) is missing only in Hdep-prov1. There are also several additional genes compared to HF2, such as the TBP-family, HalOD1-domain, DUF262-domain and MarR-family proteins (labeled below the Hdep-prov1 map).

### 3.6. Host Specificity and Comparative Genomics

**Figure 6:**
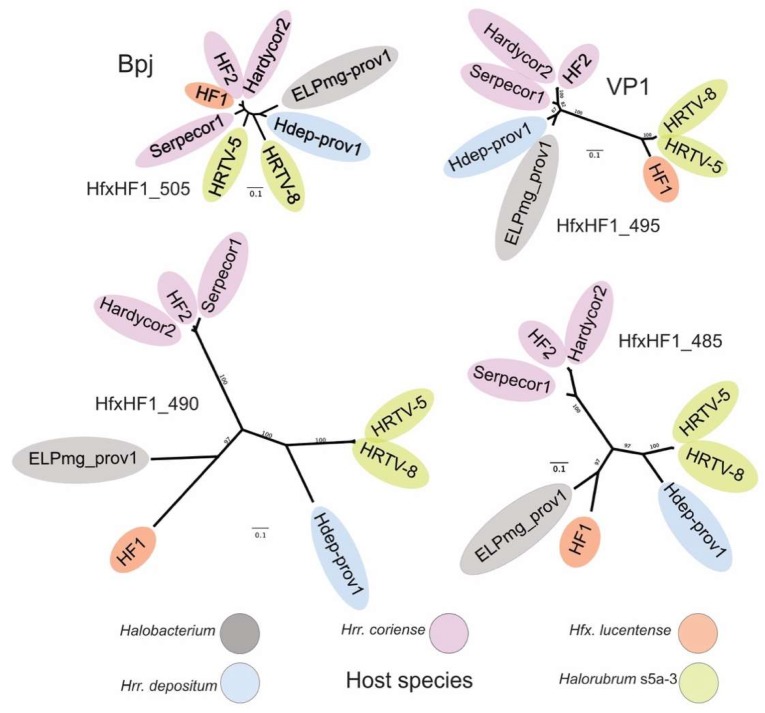
Correlation of host specificity and inferred phylogeny of variable (MDR-B) tail-proteins. Host specificity is indicated by background color, with the key shown below the trees. Homologous proteins were first aligned using MAFFT and trees inferred using PhyloML. Bootstrap values are shown near branch points and the scale bars indicate the number of expected substitutions per site. The trees have been sized so that the scale bars are equivalent. Trees are labeled by the locus tag of the HF1 protein in each homologous group, and the two top trees also have functional indications (Bpj for baseplate J family protein; VP1 for the minor virion protein). HRTV-5 and HRTV-8 were isolated on different strains of *Halorubrum* (strains s5a-3 and B2-2, respectively) but both infect three strains of *Halorubrum* (strains s5a-2, s5a-3 and SS1-3) at high EOP, indicating that they share similar RBPs [6]. The proviruses Hdep-prov1 and ELPmg-prov1 are described in the text and Table 2.

Viruses of the HF1-group are closely related but vary widely in their host specificity (Table 2, and Figure 4, right side), which should be reflected in the receptor binding proteins (RBPs) they use for attachment to their cognate host species. In caudoviruses, these are usually tail proteins that carry receptor binding domains (RBDs) [35]. Comparison of the HF1 and HF2 genomes indicated that one of two major divergent regions (MDR) carried the RBP. These MDRs were originally denoted MDR-I and -II in [3], and are now renamed MDR-B an -A, respectively, in the current study. Since MDR-A is an indel between HF1 and HF2, the most likely candidate region is MDR-B (see Figure 4), a region located just downstream of the gene encoding baseplate-J family protein (Bpj), where tail fiber genes are typically located on the genomes of similar types of viruses, such as the myohalovirus phiCh1 [5]. MDR-B encompasses three CDS, which in HF1 are HfxHF1_495 (VP1), HfxHF1_490 and _485. A comparison of the corresponding proteins in this region from all six HF1-group viruses and the two proviruses revealed that two of the three proteins within this region are strongly correlated with host specificity (Figure 6). The inferred trees of these proteins show topologies that correlate viruses with their host specificity (color-coded in Figure 6). As a comparison, a tree based on the highly conserved base-plate J family (Bpj) proteins (upper left) not only has much shorter branch lengths, but the branching pattern does not correspond to host specificity. Proteins of the HfxHF1_490 group show the typical features of a caudovirus tail fiber, with a relatively conserved N-terminal domain followed by a series of variable modules separated by glycine-rich motifs (GRMs) [36,37]). In the myohalovirus phiCh1, which has a similar virion morphology to the HF1-group as well as a similar organization of its virus morphogenesis/assembly genes [5], the RBP is encoded by the fourth gene downstream of *bpj* [38].

### 3.7. Inferred Phylogeny

**Figure 7:**
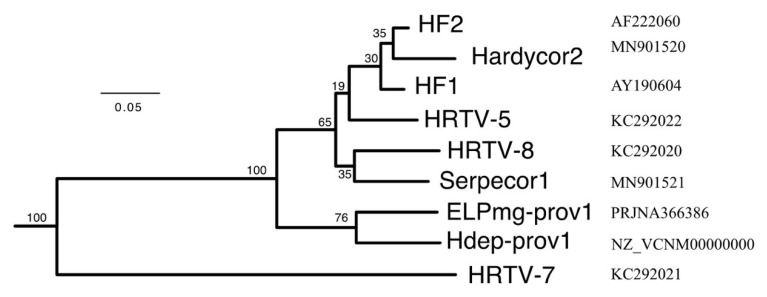
Phylogenetic tree reconstruction of viruses inferred from protein sequences using the Genome-BLAST Distance Phylogeny method (GBDP) under optimal settings (formula VICTOR *d_6_*), as implemented at the DSMZ webserver https://victor.dsmz.de. Percentage support values are shown at branch points. The branch lengths are scaled in terms of the GBDP distance formula *d_6_* [17]. Tree scale (0.05) is indicated by the bar. Accessions are given at the right. For details of strains and proviruses, see Table 2.

The protein sequences encoded by all HF1-group viruses were used to infer phylogenetic trees using the VICTOR webservice (https://victor.dsmz.de). This implements the Genome-BLAST Distance Phylogeny method (GBDP) as described in [17], and is designed to assist delineating virus taxa, particularly at the genus and species levels. A representative tree is shown in Figure 7, and shows that six of the seven viruses form a tightly clustered and strongly supported clade. HRTV-7 branches before this clade but is still specifically related to them. This analysis also included taxonomic predictions (Supplementary Table S4), which indicates they all belong to the same viral genus, and all represent different species within that genus, except for HF1 and HF2, which were placed in the same species.

## 4. Discussion

This study focused on two novel haloviruses, Hardycor2 and Serpecor1, two novel species belonging to an expanding virus group that currently includes five other viruses (HF1, HF2, HRTV-5, HRTV-8 and HRTV-7) and two proviruses, Hdep-prov1 and ELPmg-prov1. Altogether, they originate from six different countries across four continents, demonstrating a broad distribution. They are all myoviruses and have linear, dsDNA genomes of around 69–77 kb with long TDRs. The genomes share a similar gene organization with distinct functional modules. From the left terminus (Figure 4), these include a module for DNA packaging and virion assembly, followed by a module of genes for nucleic acid metabolism, recombination and replication, and finally a long module that stretches to the other terminus but for which little can be deduced because most genes encode proteins of unknown function. However, since genes in the latter module are expressed early in infection [4] it could be speculated that they may be involved in the evasion of host defenses, altering host gene expression, regulating the lytic or temperate pathways, and maintenance of the provirus in the host genome. All members carry a gene for DNA polymerase (family B). Some members have wide host ranges (HF1, HRTV-7), with HF1 being shown to infect species of three different genera: *Haloferax*, *Halobacterium* and *Haloarcula* [1], including *Hfx. volcanii*. A proposal to classify this virus group as a new genus, the *Haloferacalesvirus*, has been submitted to the ICTV. 

Comparative analyses of HF1-group genomes allowed many improvements in their annotation, particularly for CDSs that were previously doubtful but were found in the present study to be conserved across most or all members. Sequence errors in HF1 and HF2 could also be detected and corrected. Some of the non-coding, conserved features, such as tRNA-like sequences and the hammerhead ribozyme, will need further study to understand their functions. The highly conserved but partial tRNA-Thr sequence (e.g., HfxHF1_450) has been shown by the examples of two proviruses (Hdep-prov1 and ELPmg-prov1) to be the virus *attP* element used to integrate into a homologous host tRNA, which also demonstrates that HF1-group viruses can be temperate, as suggested by their conserved site-specific integrase. It is curious then, that the plaques of HF1 and HF2 are clear and not turbid [1], that infected cultures show good lysis and high virus titres, and that provirus forms of these viruses have not been detected [3], indicating a lytic lifestyle. There may be many explanations for this, including biases in isolation methods, incompatible *attP* sequences (e.g., as appears to be the case with HF1 and *Hfx. lucentense*; data not shown), or laboratory culture conditions. Further work needs to be done to understand this issue, perhaps using *Hrr. depositum* and its provirus, or by engineering genetically tractable strains such as *Hbt. salinarum* or *Hfx. volcanii*.

Prokaryotic viruses have many strategies for evading host defences [39] but the extensive avoidance of palindromic sequence motifs seen in the genomes of the HF1-group is remarkable, and indicative of a strong, purifying selection imposed by diverse sequence-specific defenses of their host species. The absence of CTAG is now extended to the genomes of eight members, and Hdep-prov1 has only three sites. This is consistent with the CTAG modification methylase Zim being widely distributed among haloarchaea, and CTAG being underrepresented in their genomes [21]. The participation of the Zim methylase in the restriction of introduced DNA has been confirmed experimentally in *Hfx. volcanii* [22]. GATC is also absent in seven HF1-group virus isolates and significantly under-represented in both proviruses. A restriction system in *Hfx. volcanii* has been shown to target *dam*-methylated (Gm^6^ATC) DNA [40] and can be circumvented either by using non-methylated DNA or abolished by deletion of the gene *mrr* encoding Mrr restriction endonuclease [20]. Curiously, the motif GATC is commonly over-represented in haloarchaeal genomes, particularly those that carry the cognate methylase [21]. This could be related to the presence of a phosphorothioation-based antiviral system in archaea that modifies DNA at the motif GATC [23]. HRTV-7 also lacks the motif AGCT, which presumably represents another common R–M target used by the host species of this virus. More subtle, non-palindromic motifs may also be avoided by HF1-group viruses but were not focused upon in this study. For example, the BREX defence system [41] is widespread among haloarchaea [42] and uses non-palindromic 6mer-motifs to recognize foreign DNA.

Most HF1-group members carry a N-6 methylase gene. In the myohalovirus phiCh1, the function of its N-6 methylase gene has been well studied [43,44]. The phiCh1 gene is expressed only late in infection, and modifies a proportion of sites on genomes that have not yet been packaged, the proportion varying between 5–50% depending upon growth conditions. It is hypothesized that this strategy allows the virus to maintain palindromic sites but produces a diversity of progeny that can evade varying types of attack; the first where host recognition of virus DNA is blocked by target methylation, and the second where recognition requires methylated target motifs. The phiCh1 methylase modifies GATC motifs, but since these are not present in HF1-group viruses, their methylases must target other motifs.

When the genomes of HF1 and HF2 were first compared, two major divergent regions were described, MDR-I and -II [3]. In the present study, these have been renamed MDR-B and -A, respectively (indicated in Figure 4 below the map of Hardycor2). The more recent members of the HF1-group have revealed another region of high variability, MDR-C, located near *attP* and often including a long gene specifying an N-6 DNA methylase (e.g., HfxHF1_460). The methylase gene may be present or absent, and, if present, can be in either orientation (compare HF1 to Hardycor2), indicating a region able to tolerate considerable genetic flexibility. In the provirus Hdep-prov1, this region is extended to include not only a methylase gene but several other adjacent genes. Since they all lie close to the integration site of the virus, they could have been captured from the host chromosome during previous excision events of the provirus. The MDR-A variable region occurs upstream of the gene encoding baseplate-J family protein (BpJ), and presumably involves tail assembly genes, but these do not correlate with host specificity. MDR-B includes the genes encoding the minor virion protein VP1 (e.g., HfxHF1_495) and two genes downstream of this. The latter two genes specify proteins that correlate closely with host specificity, and these probably carry RBDs that interact with the virus receptors present on host cells.

Many predicted proteins remain designated as hypothetical, with no known function. Those located within the DNA packaging and virion assembly modules, which together span about half the genome, are likely to specify proteins involved in virion formation and cell exit, but for those present elsewhere there are few clues. Among these are many small proteins containing one or more CxxC motifs. Such motifs are indicative of interaction domains, including zinc-finger DNA-binding domains, and in a recent study of one-domain ZF micro-proteins of *Hfx. volcanii*, many were found to regulate cell activities, such as stress adaptation, biofilm formation and swarming [45]. In earlier work, Brz, a small zinc-finger protein, was shown to regulate multiple genes, including *bop*, *crtB1*, *OE3107F* and *OE3095R* [46]. A more distant example is AFV1p06, a small (59 aa) protein encoded by the thermophilic archaeal virus AFV1 [47]. AFV1p06 carries a eukaryal ZF fold, can bind DNA, and is a potential transcriptional regulator that belongs to a protein family with members in many thermophilic viruses and archaea. It is to be expected that the CxxC motif proteins of the HF1-group will reveal many unexpected functions and insights into virus–host interactions, and those members of the HF1-group with genetically tractable hosts, such as HF1 (*Haloferax*, *Halobacterium*), would be the most favorable systems for deeper study.

## Figures and Tables

**Table 1:** Sequencing details for viruses Hardycor2 and Serpecor1.

Virus	Host	Lake ^1^	Sequence Reads ^2^	Total Mb	Genome Length (bp)	G+C %	Read Coverage	Accession
Hardycor2	*Hrr. coriense*	LH	116,990	60.3	77,342	55.6	380×	MN901520
Serpecor1	*Hrr. coriense*	SL	156,543	69.7	74,196	57.0	530×	MN901521

^1^ LH, Lake Hardy; SL, Serpentine Lake. ^2^ Read length, 250 nt.

**Table 2:** Characteristics of HF1-like viruses.

Virus or Provirus	Country	Host	Length (bp)	CDS	Terminal Direct Repeat (bp)	Accession	Reference
HF1	Australia	*Hfx. lucentense*	75,898	125	306	AY190604.2	[1,3]
HF2	Australia	*Hrr. coriense*	77,672	126	306	AF222060.2	[1,3,4]
Hardycor 2	Australia	*Hrr. coriense*	77,342	125	306	MN901520.1	This study
Serpecor 1	Australia	*Hrr. coriense*	74,196	126	320	MN901521.1	This study
HRTV-5	Italy	*Halorubrum.* str. s5a-3	76,134	118	271	KC292022.1	[6,7]
HRTV-8	Thailand	*Halorubrum*. str. B2-2	74,519	124	346	KC292020.1	[6,7]
HRTV-7	Italy	*Halorubrum*. str. B2-2	69,048	105	340	KC292021.1	[6,7]
Hdep-prov1	China	*Hrr. depositum*	77,650	120	312(?) ^1^	NZ_VCNM00000000	[18]
ELPmg-prov1	USA	*Halobacterium.* spp.	77,711	117	387	^2^ PRJNA366386	[19]

^1^ the exact length of this TDR sequence is uncertain, as indicated by the question mark. ^2^ scaffold JGI12451J12833_1000001; NCBI sample ID SAMN06268794; viral cluster vc_6654.

**Table 3:** Under-represented palindromic tetramers in HF1-group virus genomes.

	Under-represented Tetramers ^1^				
Virus/provirus	CTAG	GATC	AGCT	TGCA	CATG
HF1	0	0	.	0.19	0.42
HF2	0	0	.	0.19	0.42
Serpecor1	0	0	.	0.34	0.44
Hardycor2	0	0	.	0.19	0.44
HRTV-5	0	0	.	0.27	0.35
HRTV8	0	0	.	0.45	0.37
HRTV-7	0	0	0	0.32	0.36
ELPmg-prov1	0	0.45	.	0.58	0.39
Hdep-prov1	0.02	0.45	.	0.70	0.24

^1^ under-representation calculated as Odds Markov values where they are not zero. Dots indicate normal or near-normal frequencies.

## Supplementary Materials

The following are available online at https://www.mdpi.com/2073-4425/11/4/405/s1, Table S1: Revisions to the genome sequences of HF1 and HF2, Table S2:, Absent 6-mer palindromic restriction sites in all HF1-group viruses, Table S3: Palindromic 6-mer motifs present only in one or two HF1-group viruses, Table S4: Taxonomic predictions of HF1-group viruses using the VICTOR suite of programs. Figure S1: N-6 methyltransferases encoded by HF1-group viruses and proviruses, Figure S2: Mass spectrometry results for HF2 virus proteins VP1–VP4, Figure S3: Class I and Class II intergenic repeats (IR) of halovirus HF2 [4] compared to the corresponding sequences of Hardycor2 and Serpecor1.
