# Supplementary material for: Comparative Genomics of Two New HF1-like Haloviruses
Source: Genes (Basel). 2020 Apr 8;11(4):405. doi: 10.3390/genes11040405 (PMC7230728; doi:10.3390/genes11040405)
Supplement: Supplementary file 1 [file genes-11-00405-s001.pdf]

Supplementary Materials.

Table S1. Revisions to the genome sequences of HF1 and HF2[a]

**Position in original HF1 sequence**	**Sequence change**	**Comment**
60699	insert A after	shortens the C-terminus of CDS for the tail sheath protein (HfxHF1_555); now matches that of HF2
66205	delete T	splits one long CDS into two shorter ones (HfxHF1_575 and HfxHF1_580); now matches those of HF2
**Position in original HF2 sequence**	**Sequence change**	**Comment**
50892	insert C after	extends the N-terminus of probable tail fibre protein (now designated HrrHF2_490); now matches the N-terminus of the corresponding HF1 protein.
55232	delete T	extends the N-terminus of CDS (now designated HrrHF2_515); matches the HF1 protein
69095	insert G after	joins two CDS into one long CDS representing the major capsid protein (now designated HrrHF2_590); matches the HF1 protein.
69122	Insert C after	

[a]Original HF1 and HF2 sequence accessions are AY190604.1 and AF222060.1, and the revised sequences are AY190604.2 and AF222060.2, respectively.

Table S2. Absent 6-mer palindromic restriction sites in all HF1-group viruses[1]

Motif	Known Enzyme
AACGTT	*AclI*
AAGCTT	*HindIII*
ACCGGT	*AgeI*
ACGCGT	*MluI*
AGTACT	*ScaI*
CACGTG	*AcvI*
CAGCTG	*PvuII*
CCATGG	*NcoI*
CTCGAG	*XhoI*
CTGCAG	*PstI*
GAGCTC	*SacI*
GATATC	*EcoRV*
GCATGC	*SphI*
GGCGCC	*KasI*
GGTACC	*KpnI*
GGGCCC	*ApaI*
GTATAC	*XcaI*
GTGCAC	*SnoI*
TGCGCA	*MstI*
TGTACA	*AauI*
TTGCAA	-

[1]Excluding all palindromes with GATC or CTAG. Highlighting indicates underrepresented 4-mer palindromes.

Table S3. Palindromic 6-mer motifs present only in one or two HF1-group viruses[1]

6-mer Palindrome/Motif	Enzyme	Comment
ACATGT	*PciI*	only in HRTV-7, 3 sites
AGCGCT	*FunI*	only in HRTV-7, 1 site
AGGCCT	*AatI*	only in HRTV-8, 1 site
ATCGAT	*ClaI*	only in the two proviruses, 4-6 sites each
ATGCAT	*NsiI*	only in the two proviruses, 3 sites each
CCCGGG	*SmaI*	only in HRTV-7, 8 sites
CCGCGG	*SacII*	frequent in HRTV-7; absent or rare in others
CGGCCG	*XmaIII*	only in HF2, 1 site; and Hdep-prov1, 1 site
CGTACG	*SunI*	only in Hdep-prov1, 1 site
CTATAG	-	only in Hardycor2, 1 site; and HRTV-7, 2 sites
GAATTC	*EcoRI*	only in provirus Hdep-prov1, 1 site
GACGTC	*AatII*	only in the two proviruses, 4 sites each
GCGCGC	*BssHII*	Hardycor2, 1 site; Hdep-prov1, 32 sites
GCCGGC	*NaeI*	only in HF1; 1 site
GTCGAC	*SalI*	only in the two proviruses, 13 sites each
TCATGA	*BspHI*	only in HRTV-7, 9 sites
TCCGGA	*AccIII*	only in HRTV-5, 1 site; and HRTV-8, 1 site
TCGCGA	*SpoI*	only in provirus ELPmg-prov1, 1 site
TGGCCA	*BalI*	only in the two proviruses, 1 site each
TTCGAA[2]	*BstBI*	only in Hdep-prov1, 1 site

[1]Excluding all palindromes with GATC or CTAG. Highlighting indicates underrepresented 4-mer palindromes.

[2]methylated motif in *Halobellus limi* (http://rebase.neb.com/cgi-bin/onumget?34320)

Table S4. Taxonomic predictions of HF1-group viruses using the VICTOR suite of programs[1]

Virus/Provirus	SPECIES	GENUS	FAMILY
HF1	1	1	1
HF2	1	1	1
HRTV-8	2	1	1
HRTV-5	3	1	1
HRTV-7	4	1	1
ELPmg-prov1	5	1	1
Hdep-prov1	6	1	1
Hardycor2	7	1	1
Serpecor1	8	1	1

[1]Using the VICTOR webservice at https://ggdc.dsmz.de/victor.php. GBDP Trimming D6 OPTSIL clusters. Taxon boundaries at the species, genus and family level were estimated with the OPTSIL program (Goker et al., 2009), the recommended clustering thresholds (Meier-Kolthoff and Goker, 2017) and an F value (fraction of links required for cluster fusion) of 0.5 (Meier-Kolthoff et al., 2014).

Figure S1. N-6 methyltransferases encoded by HF1-group viruses and proviruses

(a)

HF1/HF2					
HRTV-5	98				
ELPmg-prov1	94	94			
Hdep-prov1	78	78	80		
Hardycor2	16	16	16	16	

(b)

Virus	Protein source
HF1	HfxHF1_460
HF2	HrrHF2_460
HRTV-5	HRTV5_42
ELPmg-prov1	JGI12451J12833_1000001
Hdep-prov1	FGM06_RS03845
Hardycor2	HrrHc2_200

Figure S1. (**a**) similarity matrix (% aa identities) in pairwise alignments of inferred N-6 methyltransferase proteins. (**b**) Protein locus tags for the proteins used in the similarity matrix. For ELPmg-prov1, no annotated protein is available, but the assembled scaffold ID is given (available from the JGI IMG/VR website at https://img.jgi.doe.gov/vr/).

Supplementary Figure S2. **Mass spectrometry results for HF2 virus proteins VP1 – VP4.**

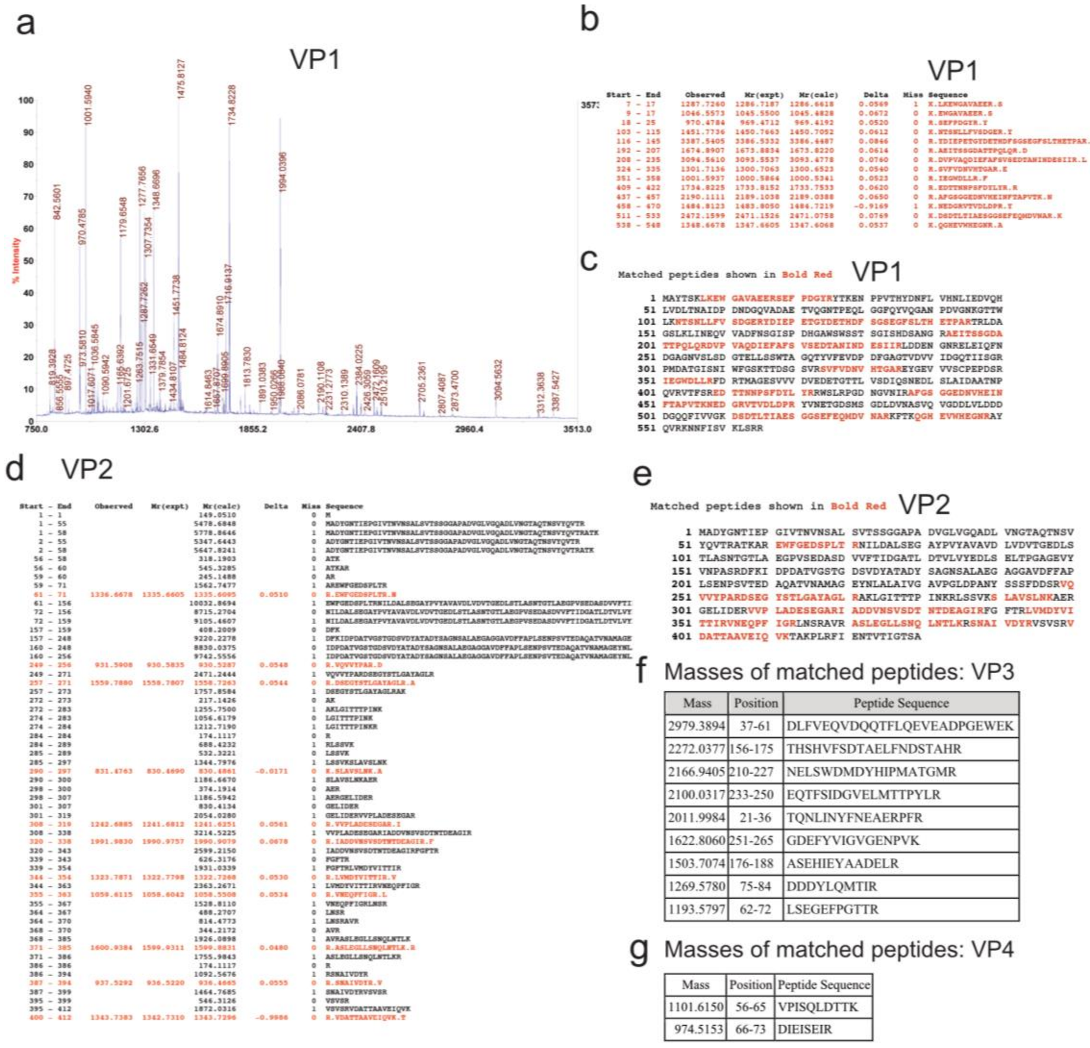

Figure S2.

(**a**) Mass spectrometry of VP1 tryptic peptides. Vertical axis, intensity; horizontal axis, m/z.
(**b**) observed and calculated tryptic peptide masses for VP1.
(**c**) positions of matched peptides in the VP1 sequence.
(**d**) observed and calculated tryptic peptide masses for VP2.
(**e**) positions of matched peptides in the VP2 amino acid sequence.
(**f**) observed tryptic peptide masses of VP3, matching peptide sequences and their positions.
(**g**) observed tryptic peptide masses of VP4, matching peptides and their positions.

Figure S3

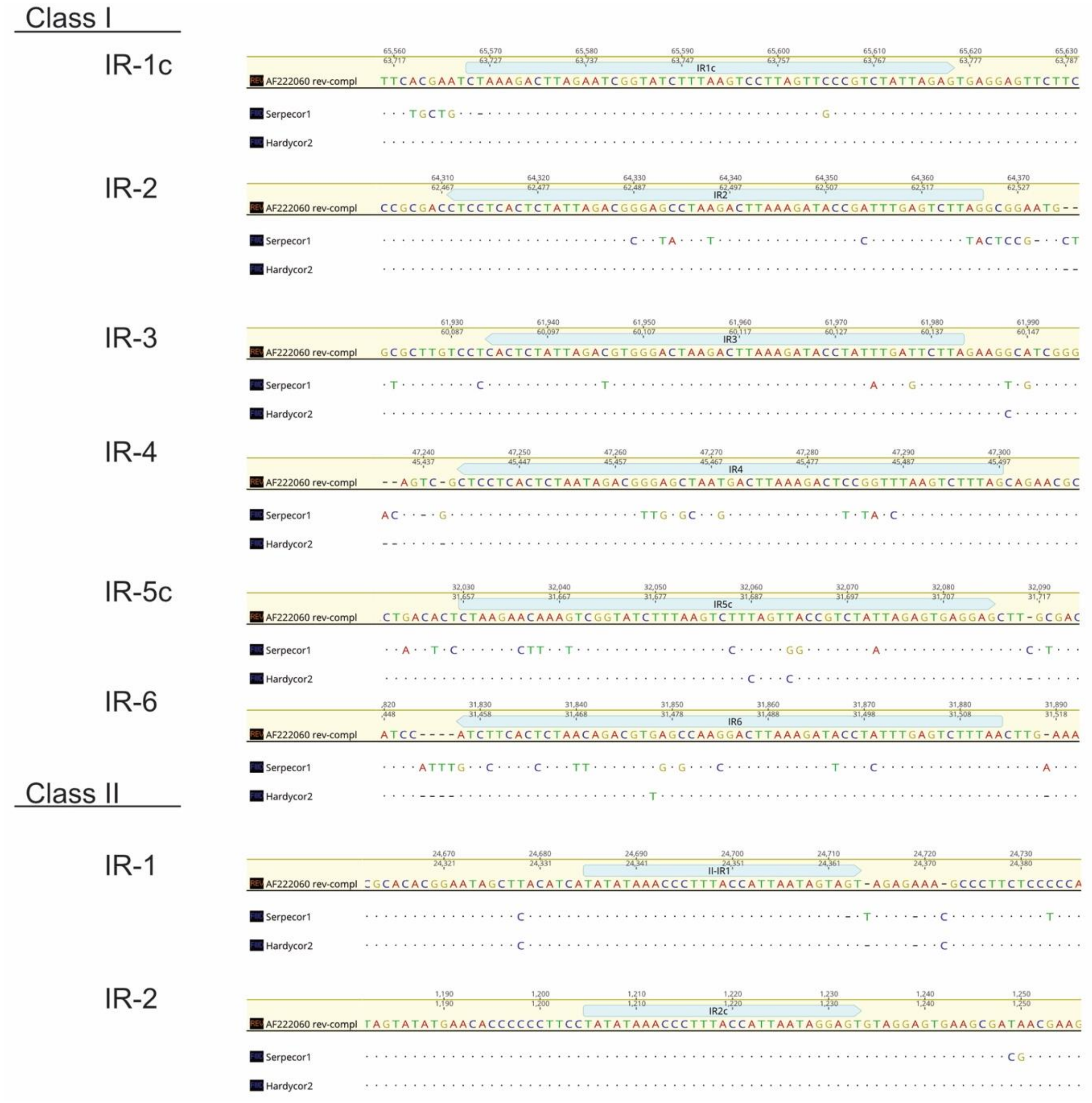

Figure S3. Class I and Class II intergenic repeats (IR) of halovirus HF2 (Tang et al., 2002) compared to the corresponding sequences of Hardycor2 and Serpecor1. The IRs are indicated by light blue arrows directly above the sequence. The dots in the alignments indicate nucleotides identical to the HF2 (AF222060) sequence.
